# Relationship of ideal cardiovascular health metrics with retinal vessel calibers and retinal nerve fiber layer thickness: a cross-sectional study

**DOI:** 10.1186/s12872-018-0922-1

**Published:** 2018-10-01

**Authors:** Qian Zhang, Dandan Wang, Anxin Wang, Shufeng Zhang, Yuesong Pan, Yang Li, Shengyun Chen, Shouling Wu, Wenbin Wei, Xingquan Zhao

**Affiliations:** 1 Department of Neurology, Beijing Tiantan Hospital, Capital Medical University, No.6 Tiantanxili, Dongcheng District, Beijing, 100050 China; 2 China National Clinical Research Center for Neurological Diseases, Beijing, China; 3 Center of Stroke, Beijing Institute for Brain Disorders, Beijing, China; 4 Beijing Key Laboratory of Translational Medicine for Cerebrovascular Disease, Beijing, China; 5 Department of Neurology, The General Hospital of Chinese People’s Armed Police Forces, Beijing, China; 6 Beijing Tongren Eye Center, Beijing Key Laboratory of Ophthalmology and Visual Science, Beijing Tongren Hospital, Capital Medical University, 1 Dong Jiao Min Lane, Beijing, 100730 China; 7 Department of Cardiology, Kailuan Hospital, Tangshan, 063000 China

**Keywords:** Ideal cardiovascular health metrics, Retinal vessel calibers, Retinal nerve fiber layer thickness

## Abstract

**Background:**

Ideal cardiovascular health (CVH) metrics have been found to be associated with subclinical vascular abnormalities. However, the relationship between ideal CVH metrics and retinal vessel calibers and retinal nerve fiber layer (RNFL) thickness in a Chinese population is unknown.

**Methods:**

We collected information on the seven ideal CVH metrics among 3376 participants aged 40 years or older from the Asymptomatic Polyvascular Abnormalities Community Study in 2012. Retinal vessel calibers and RNFL thickness were assessed by retinal photography and spectral-domain optical coherence tomography. Multivariable linear models were used to analyze the relationship between ideal CVH metrics and retinal parameters.

**Results:**

With the decreased number of ideal CVH metrics, central retinal arteriolar equivalents (CRAE) was significantly narrowed and arterio- venous ratio (AVR) significantly decreased (*p* < 0.0001). While the RNFL thickness and central retinal venous equivalents (CRVE) showed no significant changes with the decreased ideal CVH metrics. Linear regression showed that both CRAE and AVR was positively related with the number of ideal CVH metrics (regression coefficient beta: 0.806, 95% confidence interval (CI): 0.266–1.346 for CRAE (micron); and regression coefficient beta: 0.005, 95% CI: 0.002–0.009 for AVR) after adjusting for age (year), sex = male (n), education (n), average monthly income (¥) and other related risk factors.

**Conclusions:**

These findings suggested a clear positive relationship between the number of ideal CVH metrics and CRAE and AVR in Chinese population, supporting the importance of ideal health behaviors and factors in subclinical vascular abnormalities prevention.

## Background

In 2010, the American Heart Association (AHA) has proposed the concept of “ideal cardiovascular health (CVH)”, which is defined as the simultaneous presence of 4 ideal health behaviors [nonsmoking, body mass index (BMI) < 25 kg/m^2^, physical activity at goal levels, and a diet consistent with current guideline recommendations], and 3 ideal health factors [untreated total cholesterol < 200 mg/dl, untreated systolic blood pressure (SBP) < 120 mmHg and diastolic blood pressure (DBP) < 80 mmHg, and untreated fasting blood glucose < 100 mg/dl] [1]. The main aim of CVH is to improve the cardiovascular health by 20% while reducing deaths from cardiovascular diseases and stroke by 20% by 2020. This “primordial prevention” refers to preventing the initial occurrence of risk factors by adopting healthier behaviors, rather than preventing the development of a given disease, which is distinctly different from “primary prevention” [2]. Several previous studies have demonstrated a low prevalence of these ideal CVH metrics in general population and a significant inverse relationship between the number of ideal CVH metrics and the incidence of cardiovascular diseases (CVD) [3–6]. Our previous study evidences showed a similar association with CVD and stroke risk in a Chinese population [7, 8]. Ideal CVH metrics were also found to be associated with carotid intima-media thickness (CIMT) [9] and carotid plaque [10], intima media thickness (IMT), and elasticity of the abdominal aorta [11], and arterial stiffness [12]. Similar results were put forwarded by our previous study regarding the association between ideal CVH metrics and intracranial artery stenosis (ICAS) [13] and extracranial artery stenosis (ECAS) [14].Considering atherosclerosis as a diffuse and systemic disease, ideal CVH metrics may induce an effect on retinal vessels. Ogagarue et al. found that poor ideal CVH metrics were associated with wider retinal venules and narrower retinal arterioles [15]. Our previous study showed that the localized retinal nerve fiber layer (RNFL) defects were associated with previous or acute stroke [16]. We therefore examined if ideal CVH parameters were associated with central retinal arteriolar equivalents (CRAE), central retinal venous equivalents (CRVE), arterio-venous ratio (AVR) and RNFL thickness in Chinese community population.

## Methods

The Asymptomatic Polyvascular Abnormalities Community study (APAC) [17] is a community-based, prospective study to investigate the epidemiology of asymptomatic polyvascular abnormalities in Chinese adults. From June 2010 to June 2011, a total of 5440 subjects were randomly sampled from Kailuan study [7, 8, 18, 19]. A population from the previously described study who completed the baseline survey was recruited in the APAC study. The inclusion criteria were as follows: (1) aged 40 years or older; and (2) free of stroke, transient ischemic attack, and coronary disease. All the participants had undergone questionnaire assessment, clinical, laboratory, transcranial Doppler (TCD), and duplex sonography examinations during the baseline survey. During the follow-up in 2012, 3376 participants underwent examination of retinal photography and spectral-domain optical coherence tomography (SD-OCT). Data on questionnaire assessment, clinical, and laboratory examinations were also collected repeatedly like baseline examinations. The APAC study was performed according to the guidelines of Helsinki Declaration and was approved by both the Ethics Committees of the Kailuan General Hospital and Beijing Tiantan Hospital. Written informed consent was obtained from all participants and approved by the above ethics committees.

Detailed definition of ideal CVH metrics has been described before [8, 13]. Ideal smoking status was defined as having never smoked. Ideal dietary data, which was mainly based on salt intake, were defined as a consumption of < 6 g/day. Ideal physical activity was defined as moderate or vigorous physical activity for ≥80 min per week. BMI was defined as ideal if it was < 25 kg/m^2^. Blood pressure was defined as ideal if SBP was < 120 mmHg and DBP was < 80 mmHg, without the use of antihypertensives. Fasting blood glucose was defined as ideal if < 100 mg/dL and if untreated. Total cholesterol was defined as ideal if the untreated total cholesterol level was < 200 mg/dL.

Hypertension was defined as the presence of a history of hypertension, or using antihypertensive medication, or a SBP ≥ 140 mmHg, or a DBP ≥ 90 mmHg at baseline. Diabetes mellitus was defined as a self-reported history, currently treated with insulin or oral hypoglycemic agents, or fasting blood glucose level ≥ 126 mg/dl. Hyperlipidemia was defined as a self-reported history, current use of cholesterol lowering medicine, or total cholesterol level ≥ 220 mg/dl or triglyceride ≥150 mg/dl.

All the study participants underwent fundus photography to measure the retinal arteriolar and venular calibers and OCT to measure the thickness of RNFL. We used a non-mydriatic digital fundus camera (fundus camera Type CR6-45NM; Canon, Ōta, Tokyo, Japan) to take the fundus photography and the vascular calibers were measured using computer assisted quantitative assessment software (IVAN; University of Wisconsin, Madison, WI). Average CRAE and CRVE were calculated by using the Parr–Hubbard formula and were presented as CRAE or CRVE equivalent. The AVR was calculated as CRAE/CRVE.

Spectral-domain OCT images were collected from the optic nerve head, macula and adjacent retina through non-dilated pupils in a sitting position (iVue SD-OCT, Optovue Inc., Fremont, California, USA). The iVue SD-OCT used a super luminescent diode scan with a center wavelength of 840 ± 10 nm to provide high resolution images. A 6 × 6 mm^2^ raster scan was centered on the optic disc and macula.. We performed quality assurance checks. Images with failed segmentation of the RNFL, motion artifacts, poor focusing, a scan score index < 40 and images not centered on the optic disc were excluded from the assessment. Two experienced examiners scanned all study participants. Although both eyes of each study participant were detected, we used one eye’s (right eye first, if not available, we used left eye instead) mean peripapillary RNFL thickness from each participant for further analysis. Corneal curvature and spherical equivalent were adjusted when analyzing the data with ideal CVH.

Statistical analyses were performed using the SAS software (version 9.3; SAS Institute, Cary, North Carolina, USA). Continuous variables were described by means (±standard deviation) and categorical variables were described as percentages. Continuous variables including age, BMI, RNFL thickness, CRAE, CRVE and AVR were compared using analysis of variance, and categorical variables including sex, previous history of hypertension, diabetes and hyperlipidemia, family history of stroke and smoking were compared using chi-squared test in the basic characteristics comparisons. To avoid bias, we compared the key risk factors in those included population whose data were complete versus those excluded from the analyses whose data were incomplete, as shown in Table 1. Then we used a multivariable linear model to analyze the associations of CRAE and AVR with the number of ideal CVH metrics, adjusted for age (year), sex = male (n), education (n), average monthly income of every family member (¥) and family history of stroke (n). We adjusted variables of age, sex, education, income and family history because they are risk factors for retinal vessel parameters, and were associated with number of ideal CVH metrics. We also tested the interactions with age (< 60 years versus ≥60 years) and sex. All statistical tests were 2-sided, and a significant level was set as 0.05.

**Table 1:** Comparison of basic characteristics between inclusive and exclusive participants

	Entire participants (3376)	Subjects included (*n* = 3159)	Subjects excluded (*n* = 217)	*P* value
Male (n, %)	1918 (56.81%)	1793 (56.76%)	125 (57.60%)	0.83
Age, y (mean ± SD)	56.67 ± 10.33	56.17 ± 9.88	64.05 ± 13.48	< 0.0001
Previous history of disease				
hypertension(n, %)	1178 (34.89%)	1094 (34.63%)	84 (38.71%)	0.24
diabetes(n, %)	352 (10.43%)	321(10.16%)	31 (14.29%)	0.07
hyperlipidemia(n, %)	566 (16.77%)	525 (16.62%)	41 (18.89%)	0.40
Family history of stroke (n,%)	82 (2.43%)	81 (2.56%)	1 (0.46%)	0.0627
Smoking(n, %)	666 (19.73%)	634 (20.07%)	32 (14.75%)	0.0636
BMI, kg/m^2^ (mean ± SD)	24.93 ± 3.25	24.93 ± 3.22	24.93 ± 3.65	0.83
RNFL thickness, micron (mean ± SD)	101.89 ± 10.53	102.14 ± 10.37	98.16 ± 12.01	< 0.0001
CRAE, micron (mean ± SD)		153.87 ± 19.72		
CRVE, micron (mean ± SD)		232.73 ± 25.75		
AVR(mean ± SD)		0.67 ± 0.11		

## Results

We excluded 217 participants who had incomplete data on health factors or behaviors, and who had incomplete information on retinal parameters, leaving 1793 men and 1366 women for analyses in this study. The basic characteristics between the included and excluded participants were presented in Table 1. The inclusive participants were younger and had a thicker RNFL thickness. The other metrics such as gender, smoking, BMI and previous history of diseases showed no differences between the groups.

Table 2 showed the basic characteristics of participants regarding the number of ideal CVH metrics in 2012. There were significant differences in age, gender, education, and income level in participants with different number of ideal CVH metrics (*p* < 0.05).We did not observe any significant differences in the family history of stroke between different numbers of ideal CVH metrics (*P* = 0.77). Participants with a smaller number of ideal CVH metrics were more likely to have a previous history of diabetes, hypertension, or dyslipidemia.

**Table 2:** Basic characteristics of participants regarding the number of ideal cardiovascular health metrics in 2012

	Number of ideal cardiovascular health metrics							*p* value	*p* trend
	0	1	2	3	4	5	6 or 7		
Total(n)	94	393	803	878	642	278	71		
Women (%,n)	6.4 (6)	26.0 (102)	34.6 (278)	44.7 (392)	55.0 (353)	66.2 (184)	71.8 (51)	< 0.0001	< 0.0001
Age, y (mean ± SD)	55.3 ± 6.9	56.4 ± 8.7	56.6 ± 9.2	56.3 ± 10.4	56.0 ± 10.4	55.3 ± 11.3	54.7 ± 9.2	0.0023	0.12
Previous history of disease									
Diabetes (%,n)	29.8 (28)	22.4 (88)	14.5 (116)	5.9 (52)	5.3 (34)	1.1(3)	0	< 0.0001	< 0.0001
Hypertension (%,n)	55.3 (52)	54.5 (214)	42.7 (343)	33.5 (294)	23.2 (149)	12.6 (35)	9.9 (7)	< 0.0001	< 0.0001
Dyslipidemia (%,n)	35.1 (33)	30.8 (121)	21.5 (173)	14.5 (127)	7.8 (50)	6.5 (18)	4.2 (3)	< 0.0001	< 0.0001
Family history of stroke (%,n)	4.3 (4)	2.8 (11)	4.0 (32)	3.1 (27)	3.6 (23)	2.2 (6)	4.2 (3)	0.77	0.62
education (%, n)								< 0.0001	< 0.0001
High school or college	3.4 (29)	11.4 (97)	22.0 (187)	30.9 (263)	22.4 (190)	7.9 (67)	2.0 (17)		
Middle school	3.0 (50)	15.0 (251)	30.0 (495)	27.9 (465)	17.2 (287)	6.2 (103)	1.1 (18)		
Illiteracy/Primary	2.3 (15)	7.0 (45)	18.9 (121)	23.4 (150)	25.8 (165)	16.9 (108)	5.6 (36)		
average monthly income of every family member (¥)								< 0.0001	0.47
> 3000	3.5 (22)	11.6 (73)	25.4 (159)	28.4 (178)	21.1 (132)	7.7 (48)	2.4 (15)		
1000-3000	2.7 (44)	12.5 (201)	25.2 (405)	26.9 (433)	20.9 (336)	9.5 (153)	2.3 (37)		
< 1000	3.0 (28)	12.9 (119)	25.9 (239)	28.9 (267)	18.9 (174)	8.3 (77)	2.1 (19)		

*SD* standard deviation

As the number of ideal CVH metrics decreased, the CRAE became obviously narrower and AVR obviously decreased (*p* < 0.0001). While the RNFL thickness and CRVE showed no significant changes with the decreased number of ideal CVH metrics (*P* = 0.81 and 0.13, respectively), (Table 3).

**Table 3:** Mean Retinal Nerve Fiber Layer (RNFL) thickness and vessel calibers in participants with different number of ideal cardiovascular health metrics

	Number of ideal cardiovascular health metrics							*p* value	*p* trend
	0	1	2	3	4	5	6 or 7		
RNFL thickness, micron(mean ± SD)	103.03 ± 9.76	102.00 ± 10.70	101.90 ± 9.76	102.36 ± 10.95	102.11 ± 9.86	102.11 ± 11.24	102.31 ± 10.02	0.81	0.94
CRAE, micron(mean ± SD)	153.76 ± 22.02	150.45 ± 18.94	153.41 ± 19.00	153.23 ± 20.33	155.05 ± 19.59	157.85 ± 19.67	159.81 ± 18.77	< 0.0001	< 0.0001
CRVE, micron(mean ± SD)	241.05 ± 26.69	234.07 ± 25.05	232.12 ± 26.21	232.29 ± 25.86	232.47 ± 24.37	232.01 ± 27.18	231.97 ± 26.90	0.13	0.058
AVR(mean ± SD)	0.64 ± 0.10	0.65 ± 0.10	0.67 ± 0.11	0.67 ± 0.12	0.67 ± 0.10	0.69 ± 0.12	0.70 ± 0.10	< 0.0001	< 0.0001

*RNFL* retinal nerve fiber layer, *CRAE* central retinal arteriolar equivalent, *CRVE* central retinal venous equivalent, *AVR* arterio- venous ratio, *SD* standard deviation

We further used multivariable linear models to analyze the relationship between ideal CVH metrics and CRAE. Mean CRAE showed a positive relation with the number of ideal CVH metrics in the liner regression after adjusting for age (year), sex = male (n), education (n), average monthly income (¥) and other related risk factors. The results revealed for every one unit increase of the number of ideal CVH metrics, the diameter of CRAE increases by 0.806 μm. For the other variables, when the sex changes from female to male and age increases by 1 year, then the diameter of the CRAE diminishes 4.119 μm and 0.384 μm, respectively. After dividing the study group by age (year) and sex = male (n), we found a significant relationship in age < 60y subgroup and women subgroup (*P* = 0.0002 and 0.0080, respectively). However, we did not observe a significant interaction between the number of ideal health metrics and age or sex in relation to CRAE (*P* > 0.05 for both interactions), (Table 4).

**Table 4:** β Coefficients (and 95%confidence intervals) from linear models for the relationship between central retinal arteriolar equivalents and the number of ideal cardiovascular health metrics

	VIF	β	95% CI of β	*p* value	*p* interaction
Total		175.908	170.085–181.732	< 0.0001	
Number of ideal CVH metrics (n)	1.220	0.806	0.266–1.346	0.0035	
sex = male (n)	1.249	−4.119	−5.570- -2.669	< 0.0001	
age (year)	1.100	−0.384	−0.455- -0.313	< 0.0001	
education (n)	1.096				
High school or college		–	–	–	
Middle school		1.426	− 0.196- 3.048	0.08	
Illiteracy/Primary		1.702	−0.396- 3.799	0.11	
average monthly income of every family member (¥)	1.051				
> 3000		–	–	–	
1000-3000		−0.121	−1.934- 1.693	0.90	
< 1000		−0.648	−2.648- 1.351	0.53	
family history of stroke (n)	1.005	− 1.325	−5.058- 2.408	0.49	
Age (year)					0.0656
< 60y^a^					
Number of ideal CVH metrics	1.275	1.185	0.558–1.812	0.0002	
≥ 60y^a^					
Number of ideal CVH metrics	1.146	0.243	−0.885- 1.371	0.67	
Sex (n)					0.36
Men^b^					
Number of ideal CVH metrics	1.062	0.694	−0.048- 1.436	0.07	
Women^b^					
Number of ideal CVH metrics	1.018	1.089	0.285–1.894	0.0080	

*CI* confidence interval

*VIF* Variance Inflation Factor

All the coefficients without footnotes in *total part* are adjusted by other variates in *total part* when presented

^a^Adjusted for: sex = male (n), education (n), average monthly income of every family member (¥) and family history of stroke (n)

^b^Adjusted for: age(year),education (n), average monthly income of every family member (¥) and family history of stroke (n)

Next, the relationship between AVR and the number of ideal CVH metrics in a linear regression adjusted by age (year), sex = male (n), education (n), average monthly income (¥), and family history of stroke (n) was assessed. We also found a positive relation with AVR and the number of ideal CVH metrics. Also when the number of ideal CVH metrics increases by every one unit, the AVR increases by 0.005. For the other variables, when the sex changes from female to male, the AVR diminishes 0.020. While when age increases by 1 year, there is no significant change in AVR. When dividing the groups by sex = male (n) and age (year), the relationship in age < 60y subgroup was more obvious than age > 60y subgroup, and was more obvious in women group than in men subgroup, though both showed a significant meaning (*P* < 0.05), (Table 5). However, we did not observe a significant interaction between the number of ideal health metrics and age or sex in relation to AVR (*P* > 0.05 for both interactions).

**Table 5:** β Coefficients (and 95%confidence intervals) from linear models for the relationship between arterio-venous ratio and the number of ideal cardiovascular health metrics

	VIF	β	95% CI of β	*p* value	*p* interaction
Total		0.654	0.620–0.687	< 0.0001	
Number of ideal CVH metrics	1.220	0.005	0.002–0.009	0.0006	
sex = male (n)	1.249	−0.020	−0.029- -0.012	< 0.0001	
age (year)	1.100	< 0.001	−0.0003-0.0005	0.71	
education (n)	1.096				
High school or college		–	–	–	
Middle school		0.004	−0.006- 0.013	0.42	
Illiteracy/Primary		0.002	−0.010- 0.014	0.79	
average monthly income of every family member (¥)	1.051				
> 3000		–	–	–	
¥1000-3000		0.005	−0.005- 0.015	0.34	
< ¥1000		0.001	−0.010- 0.013	0.84	
family history of stroke (n)	1.005	0.001	−0.021- 0.022	0.94	
Age (year)					0.12
< 60y^a^					
Number of ideal CVH metrics	1.275	0.007	0.004–0.011	< 0.0001	
≥ 60y^a^					
Number of ideal CVH metrics	1.146	< 0.001	−0.007- 0.007	0.98	
Sex (n)					0.65
Men^b^					
Number of ideal CVH metrics	1.062	0.005	0.0002–0.0092	0.04	
Women^b^					
Number of ideal CVH metrics	1.018	0.007	0.003–0.011	0.0015	

*CI* confidence interval

*VIF* Variance Inflation Factor

All the coefficients without footnotes in *total part* are adjusted by other variates in *total part* when presented

^a^Adjusted for: sex = male (n), education (n), average monthly income of every family member (¥) and family history of stroke (n)

^b^Adjusted for: age(year),education (n), average monthly income of every family member (¥) and family history of stroke (n)

## Discussion

Our study participants with larger number of ideal CVH metrics had a significantly wider CRAE and a larger AVR in univariate analysis and in multivariable linear regression adjusted for parameters such as sex, age, education, average monthly income of every family member, and family history of stroke.

As reported before, the ideal CVH metrics are strongly associated with the risk of stroke [8]. There are already some positive conclusions from other research studies regarding the relation between cerebrovascular diseases and retinal abnormalities. Data from the Beaver Dam Eye Study showed that the retinal abnormalities, such as arteriolar narrowing, were associated with long- term cardiovascular (including stroke) mortality, especially in younger people [20, 21]. The Cooper and Lindley’s study showed that these retinal caliber changes were all related with MRI-defined subclinical cerebral infarcts and lacunar infarctions [22, 23]. Data from the Rotterdam Scan Study showed that the retinal venular dilation was related to the progression of cerebral small vessel disease [24]. RNFL is one of the most important retinal layers and is very sensitive to the abnormal tiny blood circulation. Our previous study has demonstrated that no matter whether acute or previous stroke were both significantly associated with the thinning of RNFL [16]. Investigation by Kim and his associates on 4395 Korean individuals showed that the higher prevalence of thinning of the RNFL was significantly correlated with cerebral small vessel diseases as detected by magnetic resonance imaging [25]. Thus, our study focused on the association between ideal CVH metrics and retinal abnormalities, including retinal microvascular caliber changes and retinal nerve fiber layer defects.

Ideal CVH metrics have been found to be associated with subclinical vascular abnormalities [26], including subclinical markers of carotid structure and function, such as CIMT and carotid plaque [9, 10], IMT and elasticity of the abdominal aorta [11], and arterial stiffness [12]. Our previous studies showed the association between ideal CVH metrics and ICAS [13] and ECAS [14]. Our study also showed that the thinner retinal artery diameter was significantly associated with increased common carotid artery IMT [27]. Previous investigations showed correlations between the retinal microvasculature, RNFL thickness and arterial hypertension, as well as high plasma glucose, high blood lipids and smoking [15, 28–34], all of which are components of AHA CVH metrics. Our study showed that the CRAE was decreased significantly with a smaller number of ideal CVH metrics, and so did the AVR, which was consistent with the previously published results [15]. However, this study only focused on retinal microvascular abnormalities, but RNFL thickness change as another morphological marker was not included. RNFL thickness thinning is an almost qualitative marker of abnormality because it usually does not occur in normal eyes [35]. Retinal microvascular abnormalities, however, show a marked inter-individual variability within the normal population and only a gradual transition from a normal status to a pathological status. So, we examined the relationship between RNFL thickness and ideal CVH metrics in our study. However, the thickness of RNFL showed no significant differences between the participants with different number of ideal CVH metrics. The negative results may imply that the change in RNFL thickness may be more associated with some age-related disorders or small vascular diseases, but not with the atherosclerotic diseases. The retinal arteries and veins, inversely, were more associated with atherosclerosis, which was significantly influenced by ideal CVH metrics.

Potential limitations of our study should be discussed. Firstly, our study was a cross-sectional investigation, and the study design did not allow us to draw longitudinal conclusions on a causal relationship between retinal abnormalities and ideal CVH metrics. Secondly, fundus photographs were taken but no other more detailed ocular characteristics questionnaires, and examination or secondary analysis of the fundus photography were done in our study. So, we did not discuss whether other retinopathies were associated with ideal CVH metrics, or whether other retinal diseases and metrics influenced the relationship between the retinal vessels and ideal CVH metrics. In continuation to our study, further analysis on fundus photography and the relationship between retinal abnormalities and ideal CVH metrics should be discussed in the future. Thirdly, we did not use validated dietary and physical activity questionnaires, which has been discussed in the previous studies [8, 13]. However, the included parameters did reflect the status of dietary and physical activity. Fourthly, the APAC study is not a nationally representative sample and our findings may not be generalized directly to other population in China with different educational, economic and cultural backgrounds.

## Conclusions

In conclusion, participants with a larger number of ideal CVH metrics had a significantly wider CRAE and larger AVR, supporting the importance of ideal health behaviors and factors in the prevention of retinopathy.

## Abbreviations

AHA: American Heart Association
AVR: Arterio-venous ratio
BMI: Body mass index
CIMT: Carotid intima-media thickness
CRAE: Central retinal arteriolar equivalents
CRVE: Central retinal venous equivalents
CVD: Cardiovascular diseases
CVH: Ideal cardiovascular health
DBP: Diastolic blood pressure
ECAS: Extracranial artery stenosis
ICAS: Intracranial artery stenosis
RNFL: Retinal nerve fiber layer
SBP: Systolic blood pressure
SD-OCT: Spectral-domain optical coherence tomography
TCD: Transcranial Doppler
